# A new species of *Serpinema* Yeh (1960) (Nematoda: Camallanidae) in a captive false map turtle, *Graptemys pseudogeographica pseudogeographica* (Gray, 1831) (Testudines: Emydidae), in Portugal

**DOI:** 10.1016/j.ijppaw.2026.101286

**Published:** 2026-09-08

**Authors:** João T. Cruz, Adrian Cruz, Vanessa Carvalho, Carla Maia, Ricardo Parreira, David W. Ramilo, André Pereira

**Affiliations:** a Global Health and Tropical Medicine (GHTM) LA-REAL, Instituto de Higiene e Medicina Tropical (IHMT), Universidade NOVA de Lisboa, Lisbon, Portugal; b Onevet Clínica Veterinária Vetlírios, Mem Martins, Portugal; c Superior School of Health, Protection and Animal Welfare (ESPA), Polytechnic Institute of Lusophony (IPLUSO), Lisbon, Portugal; d Clínica Veterinária Dra Vanessa Carvalho, Lisbon, Portugal; e Research in Veterinary Medicine (I-MVET), Faculty of Veterinary Medicine, Lusófona University, Lisbon University Centre, Lisbon, Portugal; f Veterinary and Animal Research Centre (CECAV), Faculty of Veterinary Medicine, Lusófona University, Lisbon University Centre, Lisbon, Portugal

**Keywords:** Camallanidae, Freshwater turtle, *Graptemys*, Integrative taxonomy, Molecular phylogeny, *Serpinema*

## Abstract

A new species of *Serpinema* Yeh (1960) (Nematoda: Camallanidae) is described from a captive false map turtle, *Graptemys pseudogeographica pseudogeographica* (Gray, 1831), admitted to a veterinary clinic in Lisbon, Portugal. *Serpinema pseudogeographicum* n. sp. is characterised by seven pairs of precloacal papillae, two pairs of adcloacal papillae and five pairs of postcloacal papillae, a combination unique among all currently valid species of the genus, together with two unequal spicules bearing a distally bifurcated right spicule, and a conspicuous lobed cuticular process on the anterior vulvar lip. Partial 18S rDNA and COI sequences provide complementary molecular support: the 18S rDNA phylogeny places *S. pseudogeographicum* n. sp. as sister to *S. cayennensis* in a well-supported lineage within *Serpinema*, whereas the COI data strongly support its recognition as a distinct species. The new species is the first nominal representative of *Serpinema* described from material recovered in Europe since *S. microcephalus* Dujardin, 1845, and its recovery from an exotic turtle host highlights both the role of the exotic pet trade as a pathway for parasite introduction and the value of opportunistic sampling during veterinary procedures for advancing knowledge of helminth diversity.

## Highlights

• A new species of *Serpinema* is described from a captive freshwater turtle in Portugal.
• A lobed cuticular process is described for the first time in the genus.
• Multivariate morphological analyses confirm its placement within *Serpinema*.
• 18S rDNA and COI provide complementary molecular support for the new species.

## Graphical abstract

**Figure:**
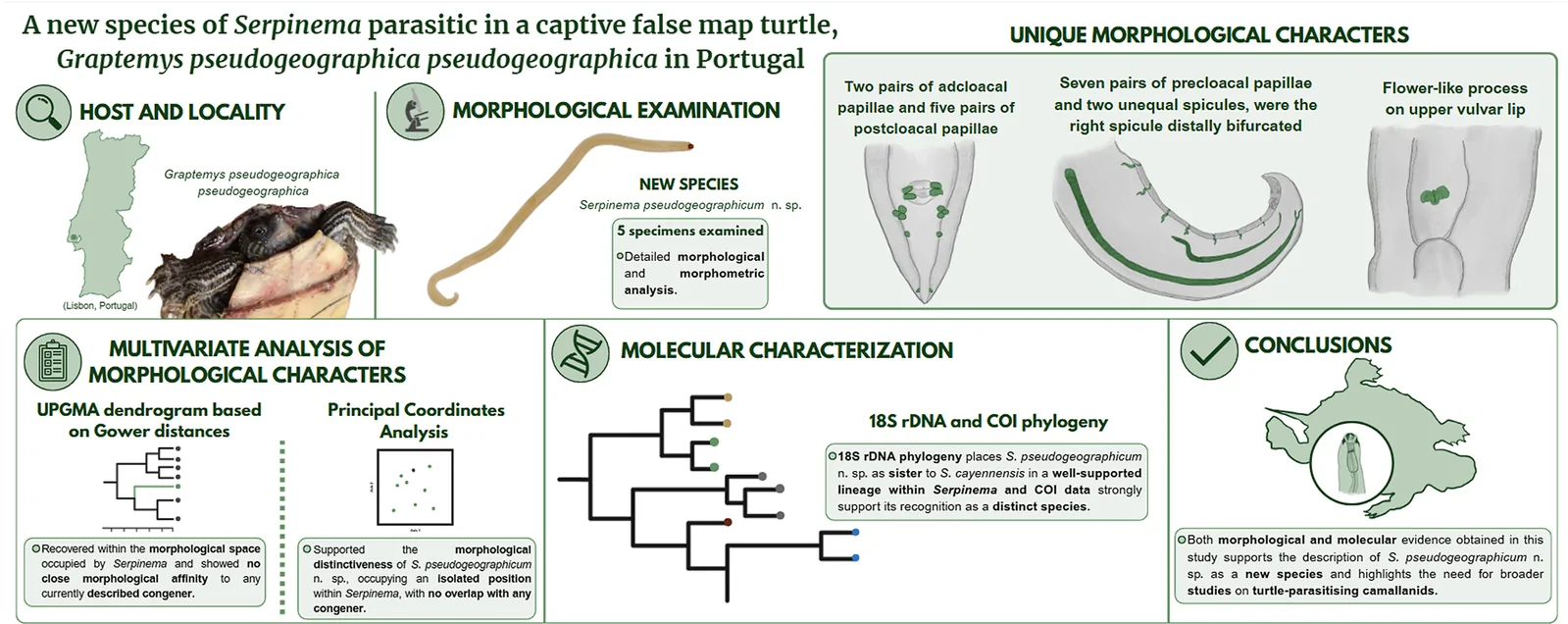

## 1 Introduction

Nematodes of the family Camallanidae Railliet and Henry, 1915 are globally distributed parasites inhabiting the digestive tract of marine and freshwater fish, amphibians and reptiles, particularly freshwater turtles (Baker, 1987; Liang-Sheng, 1960; Rigby and Rigby, 2014). Among turtle-parasitising camallanids, species have traditionally been assigned to the genus *Serpinema* Yeh, 1960 and to the species *Camallanus chelonius* Baker (1987), with additional species within *Camallanus* genus being described more recently, particularly from Australian freshwater turtles (Kuzmin et al., 2009, 2011; Rigby et al., 2008).

The genus *Serpinema* was proposed to accommodate camallanid nematodes parasitising freshwater turtles and has traditionally been distinguished from *Camallanus* by the arrangement of longitudinal ridges on the buccal capsule valves, which are grouped dorsally and ventrally with a gap between them (Anderson, 2000; Baker, 1987; Liang-Sheng, 1960). This buccal capsule configuration was also retained as a diagnostic feature in the updated key to camallanid genera proposed by Silva et al. (2026a). However, the taxonomic status of *Serpinema* has long been debated. Some authors regarded it as a subgenus or junior synonym of *Camallanus*, mainly because of similarities in buccal capsule morphology and male genital characters (Baker, 1987; Stromberg and Crites, 1974), whereas later studies revalidated *Serpinema* as a distinct genus (Harnoster et al., 2019; Sharma et al., 2002; Silva et al., 2023).

This debate was further complicated by the description of several turtle-parasitising species of *Camallanus*, especially from Australia, some of which display intermediate buccal capsule patterns (Kuzmin et al., 2009, 2011; Rigby et al., 2008). Nevertheless, phylogenetic analyses based on partial 28S rDNA showed that the Australian turtle-parasitising species of *Camallanus* form a well-supported clade separate from *Serpinema octorugatum* (Baylis and Daubney, 1923; Kuzmin et al., 2011). In addition, Harnoster et al. (2019), using 28S ribosomal DNA (rDNA), 18S rDNA and cytochrome *c* oxidase subunit I (COI) sequence data, showed that *Serpinema cayennensis* Harnoster et al., 2019 occupies a lineage distinct from the sampled species of *Camallanus*. Although no 18S rDNA or COI sequences of *Serpinema* or turtle-parasitising *Camallanus* were available for comparison, both markers indicated distant relationships between *S. cayennensis* and *Camallanus* spp. parasitising fish and amphibians, thus providing additional support for the recognition of *Serpinema* as a valid genus while also highlighting the need for broader molecular sampling to clarify generic boundaries within Camallanidae (Harnoster et al., 2019).

Species of *Serpinema* inhabit the gastrointestinal tract of freshwater turtles and may also be associated with pathological changes. For example, *S. microcephalus* (Dujardin, 1845) has been reported from the small intestine in association with eosinophilic enteritis, and aberrant migration to the pancreas has been linked to pancreatitis with necrosis and tissue destruction (Hidalgo-Vila et al., 2011; Hoseini et al., 2015).

Taking into account the updated checklist and identification key provided by Silva et al. (2026a) and the subsequent description of *S. yehi* by Silva et al. (2026b), *Serpinema* currently comprises 13 valid species: *S. amazonicum* Ribeiro (1940), *S. cayennensis*, *S. emydidium* (Mascarenhas and Müller, 2017), *S. intermedius* Hsü and Hoeppli (1931), *S. kachugae* Baylis and Daubney (1923), *S. lissemysus* Gupta and Singh (1960), *S. magathi* Sprehn, 1932, *S. microcephalus* Dujardin (1845), *S. monospiculatum* Freitas and Dobbin Júnior, 1971, *S. octorugatum* Baylis and Daubney, 1923, *S. pelliculatum* Silva et al., 2023, *S. trispinosum* Leidy, 1852; Silva et al. (2026a) and *S. yehi* Silva et al., 2026a.

In the present study, we describe a new species of *Serpinema* recovered from the false map turtle, *Graptemys pseudogeographica pseudogeographica*, in Lisbon, Portugal. The species was characterised using detailed morphological and morphometric analyses and compared with all currently valid species of *Serpinema* and selected turtle-parasitising species of *Camallanus*. Additionally, partial 18S rDNA and COI sequences were obtained to assess its phylogenetic relationships within Camallanidae and to further support its placement in *Serpinema*.

## 2 Materials and methods

### 2.1 Specimen collection

The specimens were recovered incidentally during the assisted feeding of a false map turtle, *G. p. pseudogeographica*, kept as an exotic pet in Lisbon, Portugal, and admitted to a veterinary clinic in February 2023, following a shell fracture of traumatic origin due to a dog bite. The animal had been acquired at a pet shop. Regurgitation during the clinical procedure revealed the presence of nematodes, which prompted further examination. Five specimens were collected (three females and two males) and preserved in 70% ethanol.

### 2.2 Morphological examination

Nematode specimens were cleared in Hoyer's medium 24 h prior to microscopic examination, following procedures comparable to those adopted in previous studies on *Serpinema* (Harnoster et al., 2019; Silva et al., 2023). All collected specimens were examined using a compound microscope (Olympus CX33; Olympus Corporation, Tokyo, Japan); whole-slide digital scans were obtained using a digital slide scanner (Hamamatsu NanoZoomer SQ; Hamamatsu Photonics, Hamamatsu, Japan), and all measurements and figure preparations were derived from these scans using the NDP.view2 (Hamamatsu Photonics) software integrated with the scanning system. Morphological terminology and character interpretation followed Liang-Sheng (1960) and Silva et al. (2023). Measurements are given in micrometres unless otherwise stated. Some measurements could not be performed in some specimens due to the overlap of some anatomical structures or specimen orientation.

For molecular analyses, mid-body fragments of one male and one female specimen were dissected prior to clearing; the anterior and posterior extremities, which bear the taxonomically informative structures, were preserved as vouchers for morphological identification and processed as described above, following the general strategy adopted in previous molecular studies on *Serpinema* (Harnoster et al., 2019). DNA extraction and molecular characterisation are described in the following section.

Comparative morphological and morphometric data used for the differential diagnosis were compiled for all currently valid species of *Serpinema* from original descriptions and subsequent redescriptions when available. These data included the main diagnostic characters used in species-level comparisons, namely number of spicules, number and distribution of caudal papillae, number of ridges in the buccal capsule valves, body length and width, buccal capsule dimensions, length of the middle branch of the trident, basal ring dimensions, muscular and glandular oesophagus length, presence or absence of a distal process of the right spicule, distance from the anterior extremity to the nerve ring and excretory pore, and tail length. The comparative data are summarised in Table 1. For *S. microcephalus*, the description by Caballero (1943) was adopted because earlier descriptions were poorly detailed and partly contradictory, as also noted by Ivashkin et al. (1977). For *S. magathi* the detailed description by Freitas and Dobbin Júnior (1971) was used. For *S. octorugatum* and *S. trispinosum*, the redescriptions of Sharma et al. (2002) and Moravec and Vargas-Vázquez (1998), respectively, were used. Data for all remaining species were obtained from the original published descriptions.

**Table 1:** Comparative morphological measurements (μm unless otherwise stated) of *Serpinema* species.

Character	*S. pseudogeographicum* n. sp.	*S. amazonicum*	*S. cayennensis*	*S. emydidium*	*S. intermedius*	*S. kachugae*	*S. lissemysus*	*S. magathi*	*S. microcephalus*	*S. monospiculatum*	*S. octorugatum*	*S. pelliculatum*	*S. trispinosum*	*S. yehi*
Reference	This study	Ribeiro (1940)	Harnoster et al. (2019)	Mascarenhas and Müller (2017)	Hsü and Hoeppli (1931)	Baylis and Daubney (1923)	Gupta and Singh (1960)	Freitas and Dobbin Júnior (1971)	Caballero (1943)	Freitas and Dobbin Júnior (1971)	Sharma et al. (2002)	Silva et al. (2023)	Moravec and Vargas-Vázquez (1998)	Silva et al. (2026b)
No. spicules	2	2	2	2	2	2	1	1	2	1	2	1	2	2
Distal spicule process	Present	Present	Present	Absent	Present	Absent	Absent	Present	Absent	Absent	Present	Absent	Present	Absent
No. caudal papillae pairs	14	9	13	13	14	15	18	14	12	10	14	15	15	12
No. precloacal papillae pairs	7	7	6	7	7	7	6	7	7	6	7	7	7	7
No. adcloacal papillae pairs	2	0	2	2	2	2	2	0	2	1	2	2	2	2
No. postcloacal papillae pairs	5	2	5	4	5	6	10	7	3	3	5	6	6	3
Postcloaca paillae pairs distribution	3/1/1	1/1	3/1/1	3/1	3/2	1/1/3/1	3/2/3/2	3/2/2	2/1	1/1/1	4/1	3/2/1	3/2/1	3
No. ridges in valves	9–14	17–20	8–11		10	8–10	14–16	9–11	15	10–12	6–13	9–11	16–21	10–18
Length (mm)	♂ 6.28–9.35 ♀ 12.6–16.2	♂ 11.95 ♀ 15.08–17.92	♂ 9.7–12.2 ♀ 13.7–18.5	♂ 6.55–11.4 ♀ 11.28–18.71	♂ 5.65–7.9 ♀ 11.05–12.1	♂ 10.9–14.5 ♀ 20.8–22	♂ 4.75–5 ♀ 10.5	♂ 8.34–8.93 ♀ 12.58	♂ 9–9.22 ♀ 13.85–14.6	♂ 9.55–10.92 ♀ 17.08–18.69	♂ 7.45–11.5 ♀ 7.58–21.05	♂ 6.07–8.2 ♀ 8.05–9.91	♂ 4.43–7.53 ♀ 7.47–13.35	♂ 9.72–11.73 ♀ 15.24–18
Width	♂ 240–287 ♀ 272–473	♂ 400 ♀ 530–680	♂ 332–477 ♀ 487–688	♂ 170–300 ♀ 290–400	♂ 200–230 ♀ 275–345	♂ 300–370 ♀ 450–500	♂ 210–330 ♀ 310	♂ 260–290 ♀ 290–360	♂ 236–273 ♀ 345–400	♂ 230–260 ♀ 300–360	♂ 225–375 ♀ 275–675	♂ 173–240 ♀ 227–333	♂ 163–272 ♀ 286–326	♂ 267–387 ♀ 413–507
Buccal capsule (length × width)	♂ 125–127 ×113–120 ♀ 139–164 ×132–142	♂ 180×170 ♀ 226–239 ×204–208	♂ 129–150 ×170–203 ♀ 121–179 ×213–245	♂ 128–148 ×148–178 ♀ 145–175 ×188–213	♂ 140–160 ×130–150 ♀ 165–175 ×110–130	♂ 140–160 ×110–130 ♀ 140–160 ×110–130	♂ 110–130 ×130–140 ♀ 120×140	♂ 119–133×97 ♀ 144×108	♂ 112–116 ×136–140 ♀ 120–132 ×168–176	♂ 134–144 ×108–112 ♀ 173–198 ×115–137	♂ 130–150 ×125–150 ♀ 145–190 ×140–195	♂ 95–109 ×60–91 ♀ 97–119 ×84–100	♂ 105–132 ×150–165 ♀ 144–150 ×189–195	♂ 118–129 ×143–162 ♀ 132–160 ×182–199
Trident middle branch length	♂ 89–90 ♀ 100–116	♂ 69 ♀ 78–87	♂ 105–138 ♀ 104–187		♂ 60–85 ♀ 100–120	♂ 80–100 ♀ 80–100	♂ 50–100 ♀ 50–110	♂ 61 ♀ 51	♂ 78 ♀ 56–60	♂ 72–76 ♀ 72–76	♂ 50–90 ♀ 65–105	♂ 48–69 ♀ 48–80	♂ 87–105 ♀ 105–113	♂ 68–100 ♀ 82–127
Basal ring (length × width)	♂ 8–9×89–95 ♀ 9–12×82–114	♂ 43×104 ♀ 56×139	♂ 11–17 ×106–113 ♀ 13–20 ×118–150	♂ 15–20 ×83–110 ♀ 18–23 ×113–143	♂ 35–90* ♀ 82–140*	♂ 100* ♀ 100*	–	♂ 18–22 ×86–90 ♀ 18–22 ×101–108	♂ 44×44–56 ♀ 21–26 ×112–114	♂ 25–29 ×76–79 ♀ 22–29 ×72–90	♂ 15–20 ×70–100 ♀ 15–20 ×90–130	♂ 8–13 ×60–79 ♀ 10–17 ×74–88	♂ 12–15 ×78–87 ♀ 15–21 ×99–105	♂ 13–21 ×99–116 ♀ 17–22 ×119–147
Muscular oesophagus length	♂ 422 ♀ 301–430	♂ 610 ♀ 730–750	♂ 258–548 ♀ 541–620	♂ 400–520 ♀ 510–610	♂ 450–510 ♀ 520–560	♂ 540–660 ♀ 540–660	♂ 380–440 ♀ 490	♂ 410–420 ♀ 430–450	♂ 436 ♀ 436–491	♂ 500–530 ♀ 550–740	♂ 370–520 ♀ 410–620	♂ 317–363 ♀ 368–416	♂ 367–476 ♀ 422–449	♂ 435–525 ♀ 517–592
Glandular oesophagus length	♂ 696 ♀ 623–907	♂ 530 ♀ 630–650	♂ 804–926 ♀ 895–1040	♂ 470–720 ♀ 630–760	♂ 490–550 ♀ 600–690	♂ 640–990 ♀ 540–990	♂ 400–580 ♀ 400–580	♂ 660–710 ♀ 710–780	♂ 582–618 ♀ 563–673	♂ 630–710 ♀ 710–1000	♂ 495–690 ♀ 490–930	♂ 499–613 ♀ 560–699	♂ 354–558 ♀ 490–517	♂ 717–813 ♀ 803–939
Distance nerve ring–apex	♂ 247–256 ♀ 220–273	♂ 400 ♀ 426	♂ 253–335 ♀ 303–378	♂ 190–238 ♀ 225–305	♂ 160–165 ♀ 170–215	♂ 200–230 ♀ 200–230	♂ 200–220 ♀ 250	♂ 99–132 ♀ 139	♂ 160–184 ♀ 252–296	♂ 112–132 ♀ 132–165	♂ 200–240 ♀ 210–310	♂ 141–203 ♀ 171–221	♂ 195–231 ♀ 218–299	♂ 221–284 ♀ 261–304
Distance excretory pore–apex	♂ 369–394 ♀ 519–540	–	♂ 428–510 ♀ 406–555	♂ 400–463 ♀ 425–500	♂ 260–280 ♀ 270–315	♂ 500 ♀ 500	–	–	♂ 512 ♀ –	–	♂ 350–505 ♀ 380–680	♂ 304–357 ♀ 339–437	♂ 313–326 ♀ 354–394	♂ 309–485 ♀ 371–563
Tail lenght	♀ 244–308	♀ 211	–	♀ 220–320	–	–	–	♀ 220	♀ 260–291	♀ 200–230	♀ 100–325	–	–	♀ 237–544

♂ male; ♀ female; * width only.

### 2.3 Molecular analysis

DNA was extracted from these fragments using the NZY Tissue gDNA Isolation Kit (NZYTech, Lisbon, Portugal), following the methodology described in Pereira et al. (2026).

Partial fragments of the 18S rDNA and COI genes were amplified following Harnoster et al. (2019), using primers F18ScF1 (5′-ACC GCC CTA GTT CTG ACC GTA AA-3′) and F18ScR1 (5′-GGT TCA AGC CAC TGC GAT TAA AGC-3′) for 18S rDNA, and LCO1490 (5′-GGT CAA CAA ATC ATA AAG ATA TTG G-3′) and HCO2198 (5′-TAA ACT TCA GGG TGA CCA AAA AAT CA-3′) for COI. The 18S rDNA thermocycling profile consisted of 40 cycles of 95 °C for 30 s, 58 °C for 30 s and 72 °C for 90 s, with a final extension at 72 °C for 10 min; that for COI comprised an initial denaturation at 95 °C for 3 min, 40 cycles of 95 °C for 30 s, 54 °C for 30 s and 72 °C for 2 min, and a final extension at 72 °C for 7 min. PCR products were separated by electrophoresis on 1.5% agarose gel stained with GreenSafe Premium (NZYTech) and visualised under ultraviolet illumination; fragment sizes were assessed by comparison with the NZYDNA Ladder V (NZYTech). PCR products were purified and sequenced in both directions by Sanger sequencing (STAB VIDA, Caparica, Portugal). Chromatograms were inspected, edited and consensus sequences assembled using BioEdit v7.2 (Hall, 1999).

The obtained sequences were aligned with homologous sequences retrieved from GenBank, including available representatives of *Serpinema*, *Camallanus* and other camallanid genera parasitising fishes and amphibians, using the iterative G-INS-i refinement method implemented in MAFFT v7 (Katoh and Standley, 2013). *Camallanus pallidipectoris* Palumbo, Servián, Cassano and Diaz, 2024 (GenBank accession number MN936176) was excluded, as its only available sequence (18S rDNA) corresponds to a non-overlapping region of the gene relative to the sequences obtained in this study, leaving no positions in common for alignment. For the same reason, the 18S rDNA sequence of Serpinema yehi (GenBank accession number PZ113237) was not included in the 18S dataset; the COI sequence of this species (GenBank accession number PZ113265) was included in the corresponding analysis. Ambiguously aligned positions were removed using Gblocks (Castresana, 2000) with default parameters; the COI alignment was additionally edited in AliView v1.28 (Larsson, 2014) to ensure preservation of the correct reading frame. Maximum likelihood (ML) phylogenetic trees were inferred using IQ-TREE v2.4.0 (Minh et al., 2020), with the best-fit nucleotide substitution model for each dataset selected by ModelFinder (Kalyaanamoorthy et al., 2017) based on the Bayesian Information Criterion (BIC). *Spirocerca lupi* was used as the outgroup to root both trees. Branch support was assessed by bootstrap resampling (1000 replicates) and the SH-like approximate likelihood ratio test (SH-aLRT) (Guindon et al., 2010); only values ≥ 75% are shown. Trees were visualised using FigTree v1.4.4. Nucleotide sequences obtained in this study were deposited in the DDBJ/ENA/GenBank databases.

## 3 Results

### 3.1 *Serpinema pseudogeographicum* n sp.

**Type host**: False map turtle, *Graptemys pseudogeographica pseudogeographica* (Gray, 1831).

**Type locality**: Lisbon, Portugal; material recovered from a captive *G. p. pseudogeographica*.

**Site of infection**: Not determined; specimens recovered from regurgitated material.

**Material examined**: Five specimens (two males and three females).

**Type material**: Holotype (**♂**), allotype (♀)**,** and three paratypes (1 **♂** and 2 ♀), all deposited in the Faculty of Veterinary Medicine, Lusófona University, Lisbon, Portugal, under catalogue numbers SP-2026-01 to SP-2026-05.

**Molecular data**: Partial 18S rDNA and COI sequences were generated from one male and one female specimen. The 18S rDNA sequences were deposited under GenBank accession numbers LC935963 (686 bp, ♂) and LC935964 (686 bp, ♀), and the COI sequences under accession numbers LC935965 (696 bp, ♂) and LC935966 (686 bp, ♀). The specimens used for molecular analyses are deposited under catalogue numbers SP-2026-02 (♂) and SP-2026-05 (♀).

**ZooBank registration**: In accordance with Article 8.5 of the amended 2012 edition of the International Code of Zoological Nomenclature (International Commission On Zoological Nomenclature, 2012), the present work and the nomenclatural acts it contains were registered in ZooBank. The Life Science Identifier (LSID) for this publication is urn:lsid:zoobank.org:pub:742D3B44-6F76-479F-99AE-3A3E0881BDDD, and that for *S*. *pseudogeographicum* n. sp. is urn:lsid:zoobank.org:act:6E04E1B4-83E7-4517-AB3D-BA2D7D3C18C7.

**Etymology**: The specific epithet *pseudogeographicum* is a Latinized adjectival name derived from the specific and subspecific name of the type host, *G. p. pseudogeographica*. The epithet is used in the neuter singular to agree with the generic name *Serpinema*.

### 3.2 Description

**Fig. 1:**
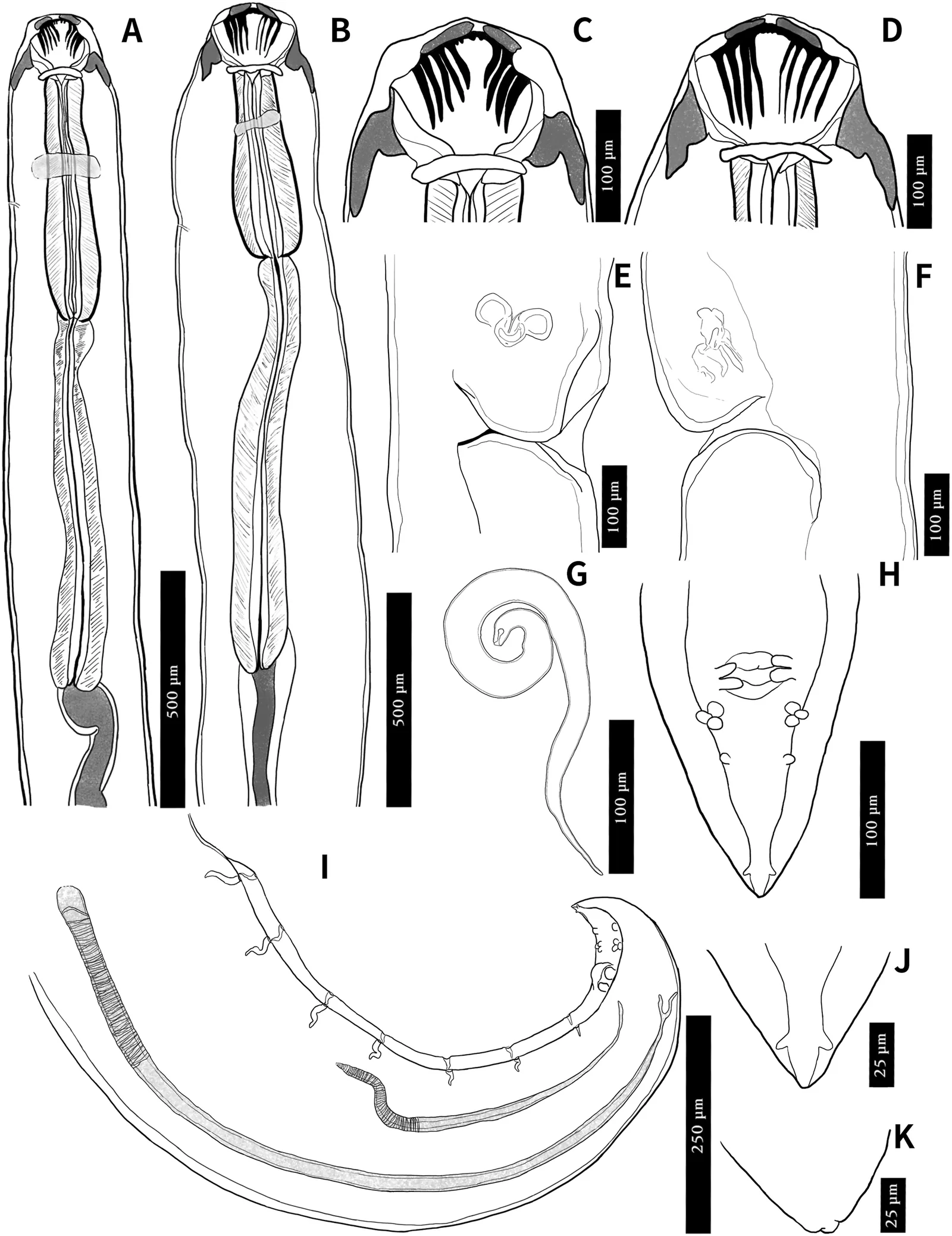
*Serpinema pseudogeographicum* n. sp., line drawings of the holotype (male) and the allotype (female). A—anterior end of body, male, lateral view; B—anterior end of body, female, lateral view; C—buccal capsule, male, lateral view; D—buccal capsule, female, lateral view; E—vulva, ventral view, showing a conspicuous lobed cuticular projection on the anterior vulvar lip; F—vulva, lateral view, showing the same lobed cuticular projection; G—first-stage larva (L1); H—posterior end of body, male, ventral view; I—posterior end of body, male, lateral view; J—last pair of postcloacal papillae near the posterior extremity of male; K—mucrons at the posterior extremity of female.

**Fig. 2:**
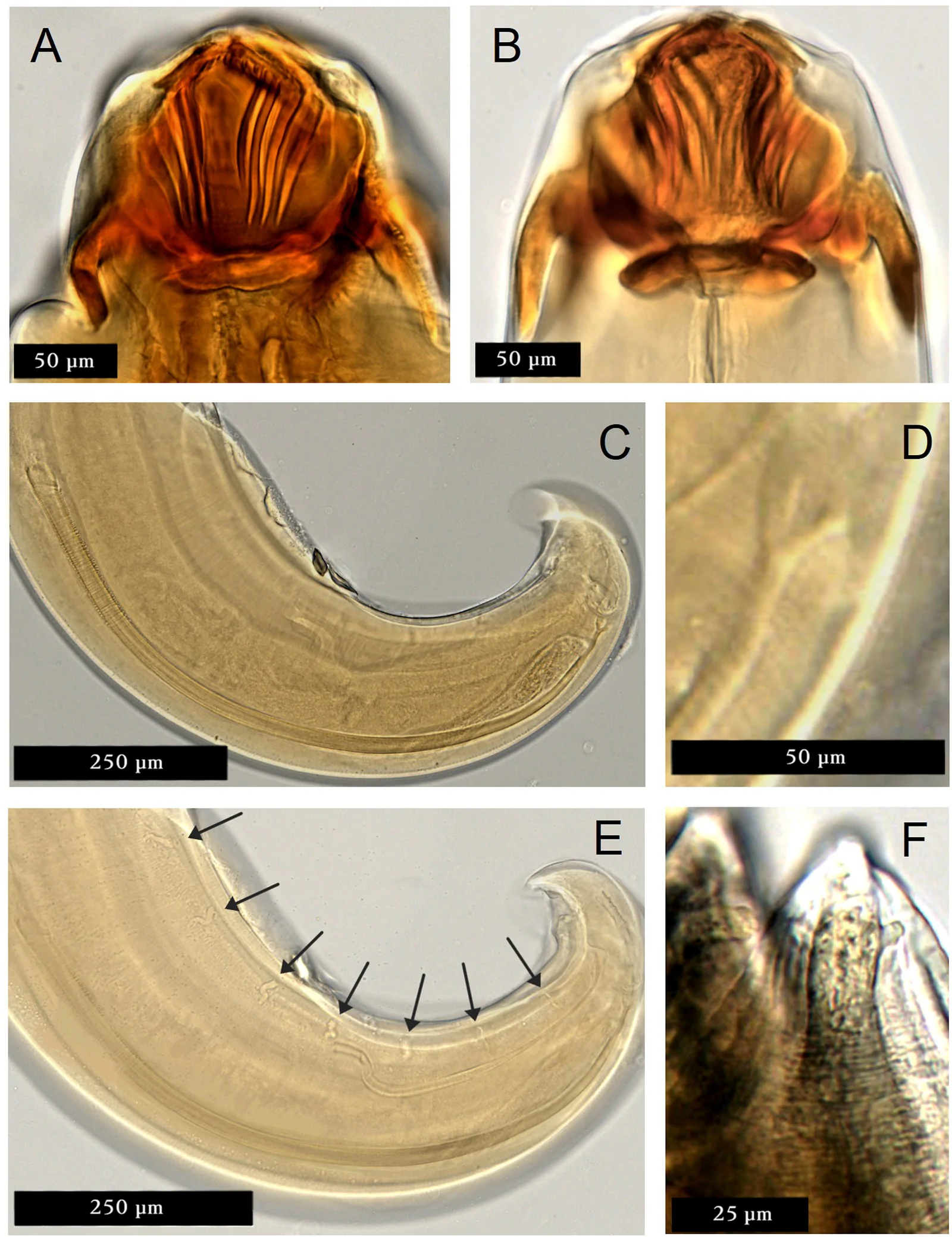
*Serpinema pseudogeographicum* n. sp., holotype male. Photomicrographs: A, B—buccal capsule, lateral view; C—posterior end of body showing the right spicule, lateral view; D—detail of the distal end of the right spicule showing the bifurcation, lateral view; E—posterior end of body, lateral view, arrows indicating the seven precloacal papillae; F—last pair of postcloacal papillae near the posterior extremity.

*General:* Medium-sized camallanid nematodes. Body slender, ventrally curved, with cuticle finely striated transversely. Specimens pinkish to reddish in life. Buccal capsule composed of two lateral valves and basal ring. Each valve bearing two pairs of elongate sclerotinized plates on its anterior region. In lateral view, these structures appeared as elongate sclerotinized bands. Buccal capsule with lateral groups of ridges separated by a central gap; each valve with 4–5 ridges on each side of central space and 0–5 small incomplete median ridges, incomplete median ridges appearing as minute denticle-like structures (Fig. 1C, D, 2A, B, 3A, B). Dorsal and ventral tridents present and equal in size, with middle and lateral branches distinct. Cephalic papillae not observed. Excretory pore posterior to nerve ring in both sexes (Fig. 1A and B).

**Fig. 3:**
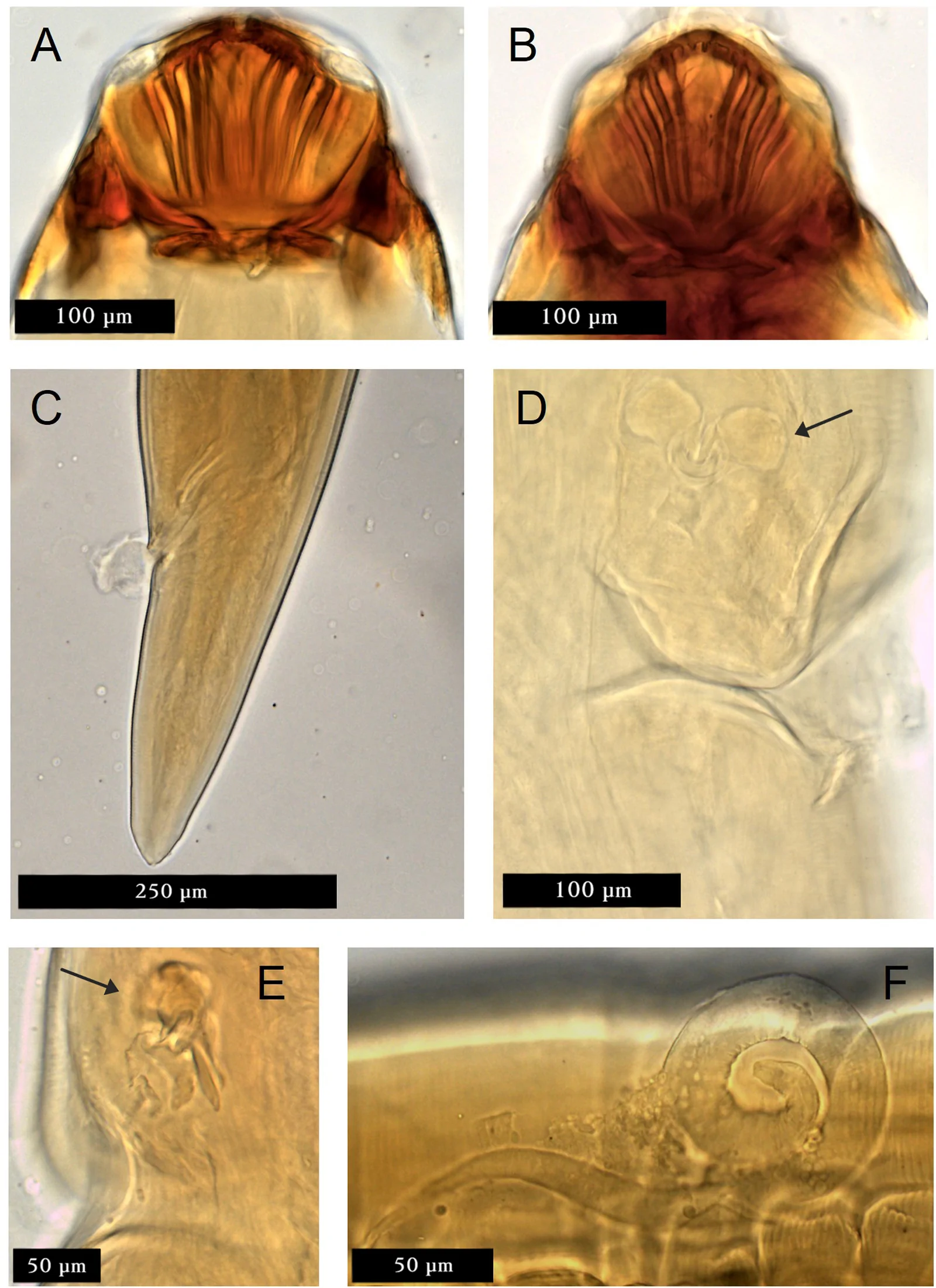
*Serpinema pseudogeographicum* n. sp., allotype female. Photomicrographs: A, B—buccal capsule, lateral view; C—posterior end of body, lateral view; D—vulvar lips, frontal view, showing a conspicuous lobed cuticular process (arrow) on the anterior vulvar lip; E—vulvar lips, lateral view, showing the same lobed cuticular process (arrow); F—first-stage larva (L1).

Measurements of the holotype and allotype are given first, followed by measurements of paratypes in parentheses when available.

*Male:* Holotype male, followed by paratype male in parentheses. Body 9.35 (6.28) mm long and 287 (240) wide (Fig. 1A). Buccal capsule 127 (125) long and 113 (120) wide. Sclerotinized plates 35.5 (33.9) long. Basal ring 9.18 (8.1) long and 95 (89) wide. Each valve with 4 (5) incomplete median ridges and 4–5 (4) lateral ridges on each side; total number of ridges 13 per valve in both specimens (Fig. 1C and 2A, B). Dorsal and ventral tridents equal in size; measures of ventral trident: middle branch 90 (89) long; lateral branches 105 long in holotype. Muscular oesophagus 488 (355) long and 119 (86) wide. Glandular oesophagus 746 (645) long and 88.5 (69.3) wide. Total oesophagus length 1234 (1000). Ratio of muscular to glandular oesophagus length 65.4% (55.0%). Nerve ring and excretory pore 256 (247) and 369 (394) from anterior extremity, respectively (Fig. 1A). Two unequal spicules (Fig. 1I and 2C, E). Right spicule 894 (900) long, bifurcated distally (Fig. 1I and 2C, D); branches of distal bifurcation 19.6 and 18.0 long in holotype. Left spicule 440 (325) long. Caudal alae 712 (500) long. Tail 183 long in paratype. Seven pairs of precloacal papillae (Figs. 1I and 2E), two pairs of adcloacal papillae and five pairs of postcloacal papillae present. One pair of adcloacal papillae situated on the anterior cloacal lip and one pair on the posterior cloacal lip. First three pairs of postcloacal papillae clustered near cloaca, fourth pair separated posteriorly, and fifth pair situated close to posterior extremity (Fig. 1H–J, 2E–F).

*Female:* Allotype female, followed by female paratypes in parentheses. Body 16.2 (12.6 in one paratype) mm long and 413 (272–473) wide (Fig. 1B). Buccal capsule 164 (139–153) long and 132 (135–142) wide. Sclerotinized plates 49.9–53.3 (40.3–44.5) long. Basal ring 11.8 (9.2–11.4) long and 114 (82.3–107) wide. Each valve with 0 (4) incomplete median ridges and 4–5 lateral ridges on each side; total number of ridges 9 (13–14) per valve (Fig. 1D and 3A, B). Dorsal and ventral tridents equal in size; measures of ventral trident: middle branch 116 (100–113) long; lateral branches 134 (128 in one paratype) long. Muscular oesophagus 430 (301–401) long and 165 (81–127) wide. Glandular oesophagus 907 (623–640) long and 119 (55.5–76.5) wide. Total oesophagus 1337 (941–1024) long. Ratio of muscular to glandular oesophagus length 47.4% (47.0–64.4%). Nerve ring and excretory pore 260 (220–273) and 519 (540 in one paratype) from anterior extremity, respectively (Fig. 1B). Vulva situated 7.78 (6.74 in one paratype) mm from posterior extremity. Vulvar lips developed but not strongly prominent; anterior lip more developed than posterior lip and bearing a conspicuous lobed cuticular process (Fig. 1E, F, 3D, E). Anus 244 (308 in one paratype) from posterior extremity. Tail ending in 3 min mucrons, 2.42 (3.61 in one paratype) long (Fig. 1K). First-stage larvae observed (Fig. 1G and 3F).

### 3.3 Remarks

Based on the comparative morphological and morphometric characters summarised in Table 1, S *pseudogeographicum* n. sp. can be distinguished from all currently valid species of *Serpinema* by a unique combination of characters, particularly the presence of two unequal spicules, the distally bifurcated right spicule, the number and distribution of male caudal papillae, and the morphology of the vulvar lips, including the conspicuous lobed cuticular projection on the anterior vulvar lip. Even disregarding spicule number, the new species can be distinguished from all congeners based on the number and distribution of the male caudal papillae, which are regarded as the most reliable characters for species differentiation within camallanid nematodes (Moravec and Vargas-Vázquez, 1998; Harnoster et al., 2019).

Silva et al. (2023) grouped species of *Serpinema* into two morphogroups according to the number of spicules. *Serpinema pseudogeographicum* n. sp. belongs to the morphogroup comprising species with two spicules and is therefore separated from the single-spicule species *S. lissemysus*, *S. magathi, S. monospiculatum* and *S. pelliculatum*. Although spicule number should be interpreted cautiously, particularly in older descriptions, these species also differ from *S. pseudogeographicum* n. sp. in the number and distribution of caudal papillae. *Serpinema lissemysus* has 18 pairs of caudal papillae, including ten postcloacal pairs; *S. magathi* has 14 pairs in total but lacks adcloacal papillae and has seven postcloacal pairs; *S. monospiculatum* has ten pairs in total, only one adcloacal pair and three postcloacal pairs; and *S. pelliculatum* has 15 pairs in total and six postcloacal pairs. In contrast, *S. pseudogeographicum* n. sp. has 14 pairs of caudal papillae, comprising seven precloacal pairs, two adcloacal pairs and five postcloacal pairs.

Among the two-spicule species, *S. pseudogeographicum* n. sp. differs from *S. emydidium*, *S. kachugae*, *S. microcephalus* and *S. yehi* by the presence of a distally bifurcated right spicule, whereas no distal process or bifurcation of the right spicule has been reported for these species. In *S. yehi*, the right spicule instead has a spatulate or leaf-like distal extremity. The new species further differs from *S. emydidium* by having 14 pairs of caudal papillae and five postcloacal pairs, instead of 13 pairs in total and four postcloacal pairs. It differs from *S. kachugae* by having five postcloacal pairs instead of six, and from *S. microcephalus* by having 14 pairs of caudal papillae and five postcloacal pairs, instead of 12 pairs in total and three postcloacal pairs. It differs from *S. yehi* by having 14 pairs of caudal papillae and five postcloacal pairs, instead of 12 pairs in total and three postcloacal pairs.

Among the two-spicule species in which a distal process or bifurcation of the right spicule has been reported, *S. pseudogeographicum* n. sp. differs from *S. amazonicum* by the presence of two pairs of adcloacal papillae and five postcloacal pairs, whereas *S. amazonicum* lacks adcloacal papillae and has only two postcloacal pairs. It differs from *S. cayennensis* by having seven precloacal pairs and 14 caudal papillae pairs in total, instead of six precloacal pairs and 13 pairs in total. It differs from *S. trispinosum* by having five postcloacal pairs and 14 caudal papillae pairs in total, whereas *S. trispinosum* has six postcloacal pairs and 15 pairs in total.

*Serpinema pseudogeographicum* n. sp. can be distinguished from *S. intermedius* and *S. octorugatum* by the arrangement of the postcloacal papillae. In the new species, the five postcloacal pairs are arranged as three clustered pairs followed by one isolated pair and one terminal pair near the posterior extremity. In *S. intermedius*, the five postcloacal pairs are arranged as three anterior pairs followed by two posterior pairs, whereas in *S. octorugatum*, four postcloacal pairs are grouped anteriorly, and one pair is situated posteriorly.

The vulvar region provides an additional diagnostic feature. In all female specimens examined, including the allotype and both female paratypes, the anterior vulvar lip bears a conspicuous lobed cuticular projection associated with the vulval opening, visible in frontal, ventral and lateral views (Fig. 1E–F, 3D–E). No comparable lobed projection has been explicitly described for other valid species of *Serpinema*. The only potentially similar condition found in the literature is the “winged” vulva reported in the original description of *S. kachugae*; however, after examination of the type material, Sharma et al. (2002) considered these wings to represent folded cuticle rather than a valid diagnostic character, noting similar folds elsewhere along the body. In the original description of *Camallanus emydidius* Mascarenhas and Müller (2017), currently *Serpinema emydidium*, no vulvar process was described; although the published photomicrograph appears to show a structure on the anterior vulvar lip, this structure is morphologically distinct from that observed in *S. pseudogeographicum* n. sp., since it is not lobed and is associated with a markedly more developed anterior vulvar lip. In *S. yehi*, the anterior vulvar lip is well developed and bears a small lateral projection near the vulvar opening. However, this projection also differs from that of *S. pseudogeographicum* n. sp. because it is not lobed and is positioned laterally near the vulvar opening, rather than forming a conspicuous lobed projection on the anterior vulvar lip.

### 3.4 Molecular characterisation and phylogenetic analyses

**Fig. 4:**
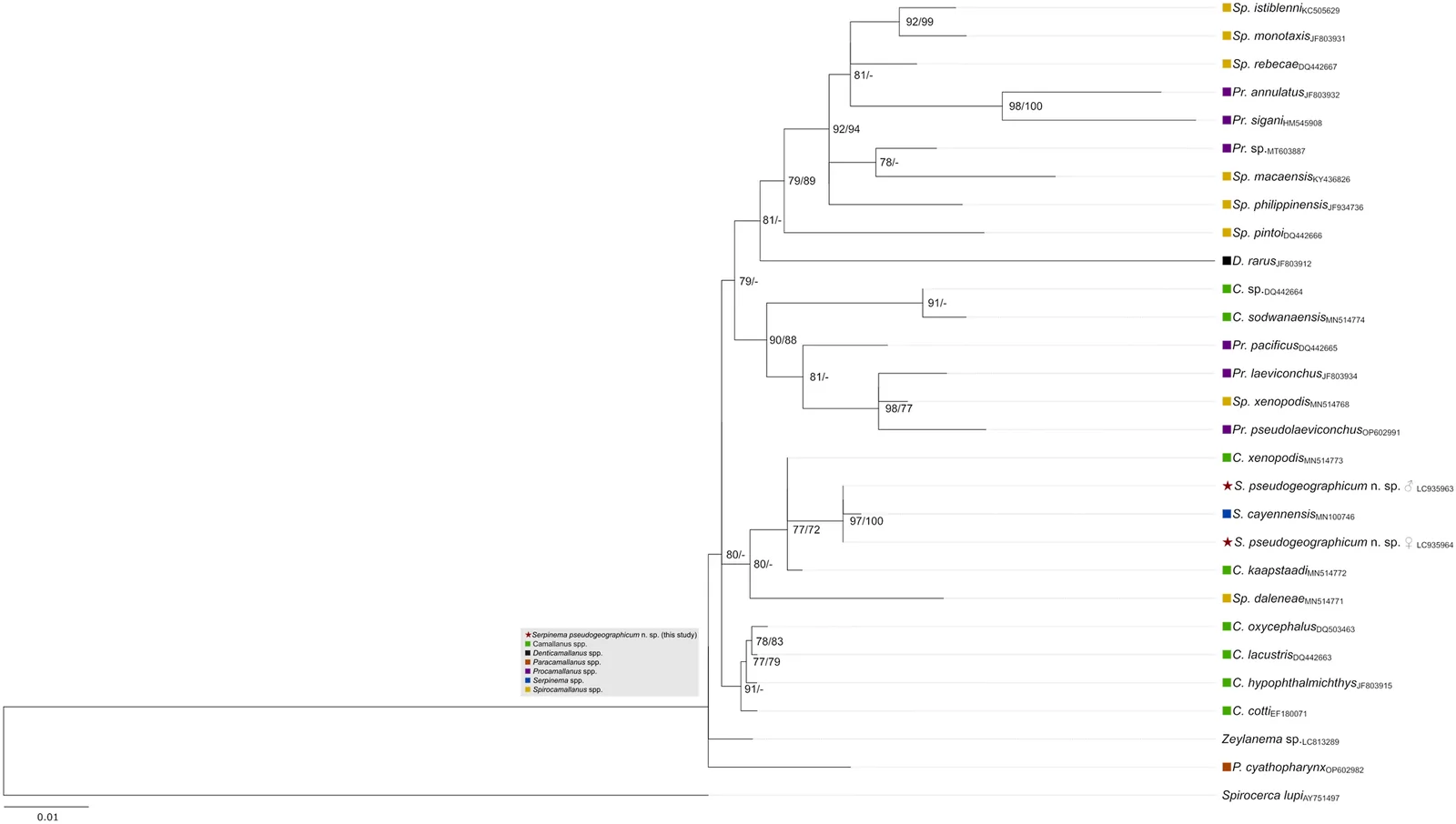
Maximum likelihood phylogenetic tree inferred from partial 18S rDNA sequences of Camallanidae (674-bp alignment). Node support values, shown at nodes as SH-aLRT/bootstrap; only values ≥ 75% are displayed. *Spirocerca lupi* was used as the outgroup. Reference sequences are labelled with genus abbreviation, species name and GenBank accession number. Sequences obtained in this study are indicated by red star symbols. Branch lengths are scaled to the number of substitutions per site.

In the 18S rDNA tree (Fig. 4), *S. pseudogeographicum* n. sp. and *S. cayennensis* clustered together in a well-supported clade (SH-aLRT 97, bootstrap 100), confirming the placement of the new species within *Serpinema*.

**Fig. 5:**
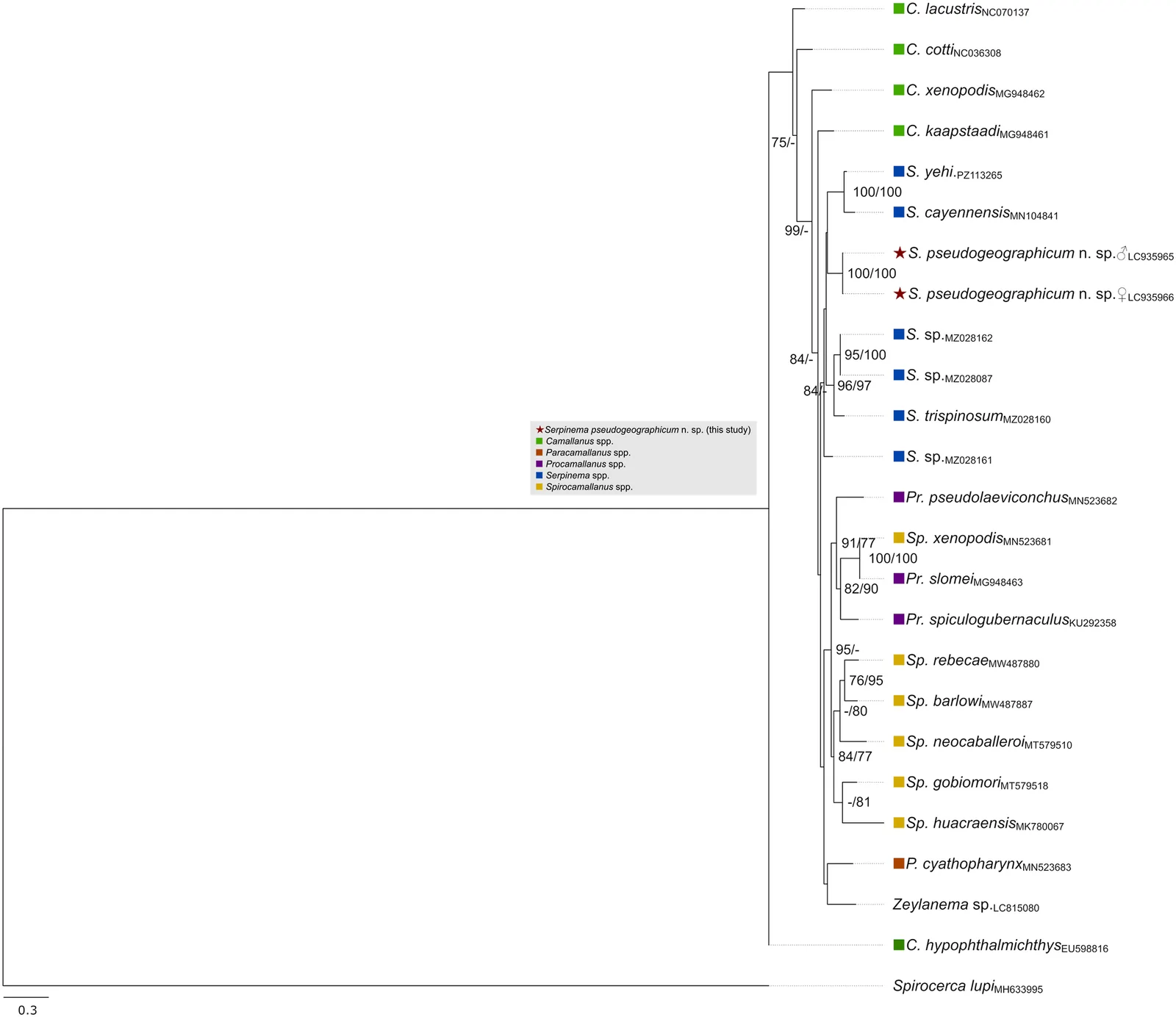
Maximum likelihood phylogenetic tree inferred from partial cytochrome *c* oxidase subunit I (COI) sequences of Camallanidae (427-bp alignment). Node support values, shown at nodes as SH-aLRT/bootstrap; only values ≥ 75% are displayed. *Spirocerca lupi* was used as the outgroup. Reference sequences are labelled with genus abbreviation, species name and GenBank accession number. Sequences obtained in this study are indicated by red star symbols. Branch lengths are scaled to the number of substitutions per site.

A comparable pattern was observed in the COI tree (Fig. 5), where the sequences of *S. pseudogeographicum* n. sp. were recovered within a clade containing all *Serpinema* spp. included in the analysis, although this genus-level grouping was supported only by the SH-aLRT test (SH-aLRT 84) and not by bootstrap. The sequences obtained from the male and female specimens formed a fully supported clade (bootstrap 100, SH-aLRT 100), clearly distinct from all other *Serpinema* spp. sequences, including *S. cayennensis*.

## 4 Discussion

The phylogenetic analyses support the recognition of the collected specimens as a distinct species. In the 18S rDNA tree (Fig. 4), the two sequences of *S. pseudogeographicum* n. sp. formed a fully supported clade, confirming the conspecificity of the analysed male and female specimens. This clade was recovered as sister to *S. cayennensis* in a well-supported lineage, placing the new species within *Serpinema* and agreeing with the molecular framework proposed by Harnoster et al. (2019).

In the COI tree (Fig. 5), the sequences of *S. pseudogeographicum* n. sp. likewise formed a fully supported clade (SH-aLRT 100, bootstrap 100), confirming the conspecificity and indicating clear genetic differentiation from all other sequences included in the analysis. All *Serpinema* sequences were recovered together, consistent with the monophyly of the genus; this genus-level grouping was, however, supported only by the SH-aLRT test and not by bootstrap, and is therefore weakly supported. Within this clade, and unlike in the 18S rDNA tree, the new species was not recovered as sister to *S. cayennensis*. The COI data thus strongly support the specific distinctiveness of the new species, whereas relationships among *Serpinema* species remain weakly resolved, consistent with the limited and taxonomically heterogeneous molecular dataset currently available for Camallanidae.

Together, the two markers offer complementary support: the 18S rDNA data primarily corroborate the generic assignment of *S. pseudogeographicum* n. sp., recovering it as sister to *S. cayennensis* within *Serpinema*, whereas the COI data primarily support its specific distinctiveness, forming a fully supported clade within a monophyletic *Serpinema*.

The placement of *S. pseudogeographicum* n. sp. in *Serpinema* is also consistent with the morphology of the buccal capsule. The new species possesses the ridge arrangement traditionally associated with *Serpinema*, in which the longitudinal ridges on the buccal capsule valves are grouped dorsally and ventrally with a gap between them. This character remains important in distinguishing *Serpinema* from morphologically similar Camallaninae genera, including *Camallanus* (Silva et al., 2026a). Nevertheless, broader molecular sampling will still be required to refine the generic boundaries within Camallanidae.

The host and recovery context of *S. pseudogeographicum* n. sp. are also noteworthy. The material was obtained from *G. p. pseudogeographica*, an exotic turtle species in Portugal, and therefore the native geographic origin of the parasite remains uncertain. It is not possible to determine whether this nematode represents a parasite introduced together with its host, a previously overlooked parasite acquired in captivity, or a species with a broader host and geographic range than is currently recognised. Consequently, the present record should be interpreted with caution from a biogeographic perspective. Because *G. p. pseudogeographica* is native to North America, a North American origin of the parasite cannot be ruled out. Several *Serpinema* spp. have been described from North American turtles, including from captive hosts (MacCallum, 1918; Magath, 1919). The new species is nonetheless distinguished from all currently valid congeners, including these North American species, by the unique combination of diagnostic characters detailed in the Remarks and Table 1, and therefore does not correspond to any previously described species. To our knowledge, *S. pseudogeographicum* n. sp. is the first nominal species of *Serpinema* described from material recovered in Europe since *S. microcephalus* was described by Dujardin (1845).

The recovery of the nematodes during regurgitation also highlights that gastrointestinal camallanids may be detected opportunistically during clinical procedures, even when the precise site of infection cannot be established.

Future studies including broader sampling of turtle-parasitising camallanids, with wider geographic representation and expanded molecular datasets encompassing additional markers or whole-genome sequencing approaches, will be essential to refine the systematics of *Serpinema* and its relationship with *Camallanus*, but the evidence currently available clearly supports the description of *S. pseudogeographicum* n. sp. as a new species.

## 5 Conclusion

The present study describes *S. pseudogeographicum* n. sp. as a new camallanid nematode recovered from a captive *G. p. pseudogeographica* in Lisbon, Portugal. The new species is supported by a unique combination of morphological characters, particularly the presence of two unequal spicules, a distally bifurcated right spicule, the number and distribution of male caudal papillae, and a conspicuous lobed cuticular projection on the anterior vulvar lip. The molecular data provide complementary evidence, with 18S rDNA supporting its placement in *Serpinema* and COI supporting its recognition as a distinct species. Although the native geographic origin of the parasite remains uncertain due to the exotic status of its host in Portugal, the morphological and molecular evidence clearly supports the description of *S. pseudogeographicum* n. sp. as a new species and highlights the need for broader taxonomic and molecular studies on turtle-parasitising camallanids.

## CRediT authorship contribution statement

**João T. Cruz:** Writing – review & editing, Writing – original draft, Visualization, Methodology, Formal analysis, Data curation. **Adrian Cruz:** Visualization, Resources, Formal analysis. **Vanessa Carvalho:** Resources. **Carla Maia:** Writing – review & editing. **Ricardo Parreira:** Writing – review & editing, Methodology. **David W. Ramilo:** Writing – review & editing, Visualization, Supervision, Methodology, Conceptualization. **André Pereira:** Writing – review & editing, Writing – original draft, Visualization, Validation, Supervision, Methodology, Formal analysis, Data curation, Conceptualization.

## Funding

This work was supported by the Veterinary and Animal Research Centre (CECAV) [UID/00772/2025; https://doi.org/10.54499/UID/00772/2025].

## Conflict interest

The authors declare that they have no known competing financial interests or personal relationships that could have appeared to influence the work reported in this paper.
